# Classification of Epidemic Community-Acquired Methicillin-Resistant *Staphylococcus aureus* by Anatomical Site of Isolation

**DOI:** 10.1155/2014/904283

**Published:** 2014-05-05

**Authors:** Jill C. Roberts

**Affiliations:** Department of Environmental and Occupational Health, University of South Florida College of Public Health, MDC 56, Tampa, FL 33612, USA

## Abstract

Methicillin-resistant *Staphylococcus aureus* contributes significantly to cost, morbidity, and mortality due to infectious disease. We surveyed community-associated MRSA isolates to determine which strains were present within anatomical sites of interest. The most likely sources of MRSA among anatomic sites swabbed were wounds followed by the nasal cavity. The USA 300 MRSA strain was most commonly isolated among wound infections while nasal swabs largely yielded USA 100 MRSA. The frequency of isolation of USA 100 amongst community-associated strains is clinically significant as this strain is often correlated with invasive disease, exhibits broad antibiotic resistance, and has been considered to be hospital associated. The potential of USA 100 to cause serious disease and the frequency of its isolation suggest an important reservoir for opportunistic infection. These data demonstrate that MRSA epidemic clones are widespread among the community.

## 1. Background

An epidemic community-associated methicillin-resistant* Staphylococcus aureus* (CA-MRSA) strain designated USA 300 has been isolated from infections in several locations within the United States [1–9]. The USA 300 epidemic clone has been associated with serious community-acquired disease, often in patients who lack the traditional risk factors for MRSA infections including hospitalization, concurrent disease, and intravenous drug use [10–12]. USA 300 carries the Panton-Valentine leukocidin (PVL) toxin genes among other virulence factors, which may contribute to its ability to cause serious disease including primary skin infections and necrotizing pneumonia. Recently, USA 300 has also been identified as a common cause of community-associated infections in Florida [9].

MRSA infections are a major public health concern due to the associated morbidity, mortality, cost of care, and limited treatment options [13]. Primary skin infections caused by USA 300 can be spread readily among groups engaging in contact sports [1, 6]. Diseases such as necrotizing pneumonia caused by PVL-positive strains, such as USA 300, have a high mortality rate as demonstrated by a study of 23 patients in which 61% of those patients succumbed to the disease [14]. The cost of care for infections caused by methicillin-resistant strains of* S. aureus* is approximately twice that of infections caused by susceptible* S. aureus* strains primarily due to greater inpatient hospital costs [15]. Most patients with skin infections are treated with trimethoprim/sulfamethoxazole alone or in combination with a second drug such as cephalexin and/or clindamycin. However, recent data suggests that resistance to these antimicrobials among community MRSA is increasing [16].

The Centers for Disease Control and Prevention (CDC) has applied the designation USA 100-800 and USA 1000-1100 to MRSA strains commonly found in the United States [7]. Pulsotypes for epidemic clones historically associated with hospital-associated infections include USA 100, USA 200, USA 500, USA 600, and USA 800 [7]. The epidemic clone USA 100 is associated with invasive disease in hospitalized patients and in each case where MRSA has become resistant to vancomycin, resulting in VRSA, USA 100 has been the strain involved [17]. The USA 200 clone is most notable for its ability to cause toxic shock as it possesses the TSST-1 or toxic shock toxin genes [18]. Interestingly, data on nasal carriage of USA 200 demonstrated a significant drop in percentage from the 2001-2002 to the 2003-2004 time periods for reasons that are unknown [19]. The USA 500 epidemic clone is most notable as the progenitor strain of USA 300. USA 500 lacks the PVL (Panton-Valentine leukocidin) genes and mobile genetic elements which have likely contributed to the high virulence and transmissibility seen in the USA 300 strains [20]. However, unlike USA 300 isolates, USA 500 isolates are more likely to be multiply resistant which may contribute to their success in the healthcare environment [19]. USA 600 has been noted among hospital isolates but has not, until recently, been reported as a cause of serious disease. However, a recent study reported a 60% mortality rate associated with blood stream infections caused by USA 600 [21]. The final hospital-associated isolate, USA 800, is highly related to USA 100 and most notable for its increased resistance to daptomycin [22]. Pulsotypes for epidemic clones historically associated with community-onset disease included USA 300 discussed above and the closely related USA 400 isolate. Although it is as a well-known community-associated strain worldwide and has historically caused serious disease in the United States, USA 400 seems to have been outcompeted by USA 300 [23]. The USA 1000 isolates occur infrequently and are usually associated with wound infections in persons who abuse drugs [7]. USA 1100 phenotype is associated with localized occurrences such as an outbreak of furunculosis reported in Alaska [7, 24]. Finally, USA 700 is associated with both community and hospital onset disease which is rarely serious.

We have previously identified the USA 300 epidemic clone among CA-MRSA isolates collected in both Florida and Washington States [9]. We identified 111 USA 300 isolates among 260 CA-MRSA collected in Florida through the use of pulsed-field gel electrophoresis (PFGE) which is considered the gold standard for* S. aureus* molecular epidemiology [7, 25–28]. Our study included isolates obtained from wounds, blood, sputa, nasal swabs, and other anatomical sites. Similar to other studies, we demonstrated that 98 of the 111 Florida USA 300 isolates were collected from wound samples [1–3, 6–10]. In the present study, we have expanded the total number of isolates collected in Florida to 291 to assess the presence of the known United States MRSA epidemic clones among a collection of Florida's CA-MRSA.

## 2. Methods

A total of 291 CA-MRSA isolates collected in Florida and submitted to the Florida Department of Health (FL DOH), Bureau of Laboratories in Tampa, were used in this study. Community-associated isolates were defined as those collected from outpatient services or within 48 hours of hospitalization. Isolates were classified as MRSA and community-associated by area hospital laboratories serving primarily central Florida including Tampa and Orlando. Some laboratories submitted more than one isolate per patient from multiple body sites. We confirmed that these isolates were identical strains and therefore only one per patient is included in this study. Control strains used for the PFGE were as follows: USA 100-USA 800 (NRS282-287, NRS123, and NRS22) and USA 1000-1100 (NRS483-4) were obtained from NARSA (http://www.narsa.net/). The PFGE marker strain, NCTC 8325 (NRS77) was used as a control on all PFGE gels and was also obtained from NARSA. The identification of all isolates as* S. aureus* and their antibiotic resistance was confirmed as previously reported [9].

PFGE was performed as previously reported for enterococcal isolates [29] with the following changes for* S. aureus*. Overnight cultures of* S. aureus* were grown in BBL Trypticase Soy Broth (Becton Dickinson, Sparks, MD). Lysostaphin (number L7386, Sigma, St. Louis, MO) was added to the EC-lysis buffer at a concentration of 3 U/mL and plugs were lysed for five hours at 37°C, followed by overnight incubation in ESP solution at 50°C. Plugs were digested in* Sma*I overnight, melted at 69°C for 10 mins, and loaded onto a 1 Seakem Gold (Cambrex BioScience, Rockland, ME) gel. PFGE was performed using a DR-II CHEF Mapper (Bio-Rad, Hercules, CA) using the following parameters: 200 V, 14°C, 5.3 s initial switch, 34.9 s final switch, and 20 hrs run time. Data was analyzed using BioNumerics (Applied Maths, St-Martens, Belgium).

Dendrograms were derived from the unweighted pair group method (UPGMA) using arithmetic averages and based on Dice coefficient.

## 3. Results 

PFGE was performed on a total of 291 CA-MRSA isolates collected in Florida. The resulting patterns were imported into the BioNumerics database and dendrograms were created as described. The majority of the isolates in our collection were assigned pulsotypes consistent with one of the previously identified epidemic clones.

### 3.1. USA 300 Epidemic Clone

**Figure 1:**
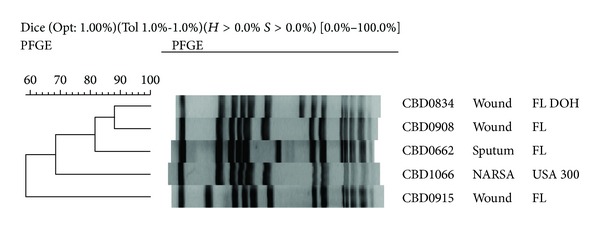
BioNumerics analysis of USA 300 epidemic clones: the NARSA USA 300 control strain (CBD1066) was used to identify most of the USA 300 epidemic clones in our collection.

Approximately half of the isolates tested possessed pulsotypes consistent with the USA 300 epidemic clone. The majority of these isolates were identified using the dendrograms generated by BioNumerics. Phylogenetic comparison was possible because the DNA fingerprints of these isolates were identical to the USA 300 control strain pulsotype (Figure 1). However, a number of previously unidentified USA 300 isolates were elucidated by visual comparison to recently published USA 300 DNA fingerprints for which there are no control strains available [23]. In this case, since there are no control strains available, it is impossible to perform phylogenetic comparison using BioNumerics. These newly recognized USA 300 isolates differed from the control strain by no more than two bands and are therefore considered identical strains (Figure 1) [30]. USA 300 accounted for 145 or nearly 50% of the CA-MRSA isolates collected in Florida, as compared to 43% of isolates in our earlier study [9]. The vast majority of these strains, 126 or 87%, were obtained from wound infections (Table 1, Figure 3), consistent with our earlier study [9]. The USA 300 epidemic clone was also rarely seen among isolates from blood, nose, sputa, and other clinical sites (Table 1, Figure 3).

### 3.2. USA 100 Epidemic Clone

**Figure 2:**
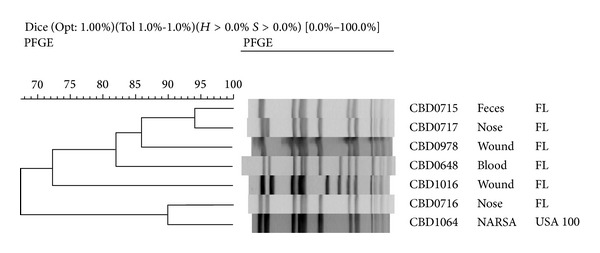
BioNumerics analysis of USA 100 epidemic clones: the NARSA USA 100 control strain (CBD1064) was used to identify most of the USA 100 epidemic clones in our collection.

**Figure 3:**
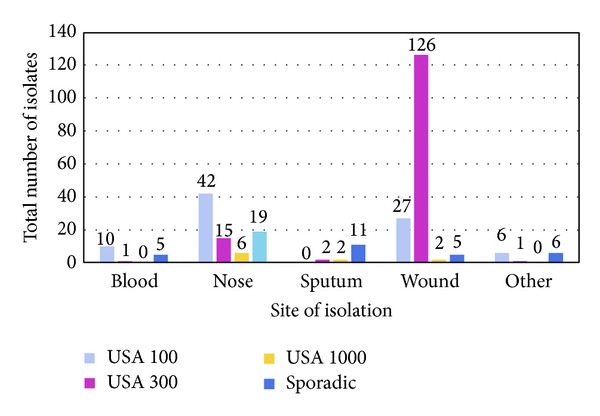
Total number of CA-MRSA isolates categorized by isolation site: CA-MRSA isolates collected from blood, nose, sputa, wound, and other clinical sites were characterized by PFGE.

In contrast to publications suggesting that USA 100 epidemic clone is a hospital-acquired strain, isolates closely related to the USA 100 control strain accounted for 29% of the CA-MRSA isolates collected in Florida (Table 1) [7]. A total of six different pulsotypes, which differed by one to two bands from the USA 100 control strain, were identified among the Florida isolates (Figure 2). While nearly 32% of these strains were cultured from wound infections, the majority of the isolates (49%) were from nasal swabs (Figures 2 and 3).

### 3.3. Other Pulsotypes Identified among CA-MRSA Isolates

PFGE analysis identified one isolate each with 100% identity to the USA 500 and USA 600 control strains (Table 1). BioNumerics analysis also demonstrated three isolates with 100% identity to the USA 800 control strain (Table 1). Further analysis of these isolates was not performed as they were rare among Florida CA-MRSA. Interestingly, 10 isolates matched by visual comparison to the published PFGE pattern for a newly described epidemic clone, USA 1000 (Table 1) [31]. The epidemic clones USA 200, USA 400, and USA 1100 were not present among the Florida isolates. The remaining isolates were categorized as sporadic as they possess pulsotypes that are unique to each isolate with no known match.

## 4. Discussion 

In the present study we demonstrate that the USA 300 epidemic clone is well established in the state of Florida, accounting for nearly 50% of all CA-MRSA isolates collected. Consistent with other studies [10–12], we demonstrated that USA 300 is the most prominent strain among isolates collected from wound infections. These data suggest that the USA 300 clone is particularly well adapted for spreading in the community environment. The potential of this highly virulent strain to cause disease in otherwise healthy individuals, along with its propensity to spread rapidly in the community environment, is a cause for concern.

Historically, MRSA isolates were well known to be multidrug resistant. And, in addition to *β*-lactam resistance, hospital-acquired isolates are frequently resistant to other classes of antibiotics. Therefore, treatment of MRSA infections has often involved the use of vancomycin. The use of vancomycin to treat MRSA infections provides selective pressure for the emergence of vancomycin-resistant* S. aureus* (VRSA) and vancomycin-intermediate* S. aureus* (VISA). However, the USA 300 epidemic clone, while being resistant to oxacillin, lacks the antibiotic resistance of the hospital strains. In a study of 187 USA 300 isolates, 100% were susceptible to chloramphenicol, gentamicin, linezolid, quinupristin-dalfopristin, and trimethoprim-sulfamethoxazole [23].

The large number of USA 100 isolates identified among the Florida CA-MRSA in the present study was unexpected since this strain was previously characterized as a hospital-acquired isolate [7]. Furthermore, national surveys of nasal colonization have reported USA 200 as the most common nasal colonizer [19]. This finding would suggest that the USA 100 clone has moved from the hospital to the community environment, but it has not been associated with any disease etiology. The majority of the USA 100 strains herein were isolated from nasal swabs, suggesting that this strain may be involved in colonization as opposed to disease. Further evidence for a role in colonization may be the fact that these isolates do not carry the PVL genes, suggesting that they lack the virulence of other MRSA epidemic clones. Regardless, many USA 100 isolates were cultured from wound sites (Table 1) possibly because most MRSA carriers will autoinfect themselves with the same isolates that they harbor [32–34]. Therefore, periodic monitoring of the rates of nasal carriage, followed by appropriate treatment to eliminate strains, may be required to stop the spread of the epidemic clone in the community environment. Although the isolates used in this study were collected from individuals in the community, it is unknown if these persons have a history of prior hospitalization. Regardless, the USA 100 strain has become well established in the community environment.

As expected, most of the remaining epidemic clones, including USA 200 and USA 500, were absent or present only in small numbers in the community. However, a recently described clone, USA 1000, was identified in 10 isolates in our study. This clone is unique in that it is rarely involved in colonization or disease except among intravenous drug users [31]. Consultations with physicians who treated these infections confirmed that in each case a history of drug use was present (personal communication). This unusual strain should therefore be considered as a possible cause of infection in this high-risk group. Similar to USA 300, USA 1000 is resistant to *β*-lactam antibiotics but many other treatment options remain.

## 5. Conclusions

The present study has demonstrated that the highly virulent USA 300 epidemic CA-MRSA strain is widespread in Florida. Florida healthcare practitioners should consider CA-MRSA as a possible cause of cases of primary skin infections including necrotizing fasciitis and as a cause of necrotizing pneumonia, especially among otherwise healthy individuals. This study has also demonstrated that the USA 100 isolate, commonly considered a hospital-acquired strain, is well established in the community in Florida. Treatment of this strain is complicated as it is multidrug resistant and sensitivity data are required. The practice of prescreening patients upon admission in some Florida hospitals followed by MRSA treatment is highly recommended to prevent invasive disease caused by USA 100.

## 6. Limitations of the Study

A limitation of this study was the use of blinded samples, required due to the nature of the funding used, wherein the clinical information from the patients was not available. The number and nature of isolates forwarded for analysis were at the discretion of the submitting laboratories within the guidelines generally accepted for community associated infections as described. The role of nosocomial spread and exact geographic location within the state of Florida of the isolates is therefore unknown to the authors of this study. Future study should include the geographic information, patient history, and powered study design to estimate the incidence of these isolates among Florida MRSA.

## Figures and Tables

**Table 1:** Summary of results for all isolates.

Source of isolates	Number of isolates for each pulsotype							Total number of isolates from source
	USA 100	USA 300	USA 500	USA 600	USA 800	USA 1000	Sporadic	
Blood	10	1	0	0	0	0	5	16
Nose	42	15	1	1	1	6	19	85
Sputum	0	2	0	0	2	2	11	17
Wound	27	126	0	0	0	2	5	160
Other	6	1	0	0	0	0	6	13
Total	**85**	**145**	**1**	**1**	**3**	**10**	**46**	**291**
